# Hydrogels and Organogels for Local Anesthetic Delivery: Advances, Challenges, and Translational Perspectives

**DOI:** 10.3390/gels12010022

**Published:** 2025-12-25

**Authors:** Jong-Woan Kim, Jin-Oh Jeong, Hoon Choi

**Affiliations:** 1 Department of Anesthesiology and Pain Medicine, Seoul St. Mary’s Hospital, College of Medicine, The Catholic University of Korea, Seoul 06591, Republic of Korea; jwjw12@catholic.ac.kr; 2 Wake Forest Institute for Regenerative Medicine (WFIRM), Wake Forest School of Medicine, Winston-Salem, NC 27157, USA; jijeong@wakehealth.edu

**Keywords:** local anesthetics, hydrogels, organogels, bigels, sustained-release delivery, perioperative analgesia, topical and transdermal anesthesia, stimuli-responsive materials

## Abstract

Gel-based depots are increasingly recognized as platforms to extend the intratissue residence of local anesthetics (LAs) while reducing systemic exposure. Hydrogels, organogels, and emerging bigels represent three distinct architectures defined by their continuous phases and drug–matrix interactions. Hydrogels provide hydrated polymer networks with predictable injectability, tunable degradation, and diffusion- or stimulus-responsive release, enabling sustained analgesia in perineural, peri-incisional, intra-articular, and implant-adjacent settings. Organogels, formed by supramolecular assembly of low-molecular-weight gelators in lipids or semi-polar solvents, strongly solubilize lipophilic LA bases and enhance barrier partitioning, making them suitable for dermal, transdermal, and mucosal applications in outpatient or chronic pain care. Bigels integrate aqueous and lipid domains within biphasic matrices, improving rheology, spreadability, and dual-solubilization capacity, although their use in LA delivery remains at the formulation stage, with no validated in vivo pharmacology. This narrative review synthesizes the design principles, release mechanisms, and translational evidence across these platforms, highlighting domain-specific advantages and barriers related to mechanical robustness, sterilization, reproducibility, and regulatory feasibility. We propose a platform-level framework in which depot selection is aligned with LA chemistry, anatomical context, and clinical objectives to guide the development of workflow-compatible next-generation LA depots.

## 1. Introduction

Local anesthetics (LAs) are essential agents for providing targeted neural blockade across a broad spectrum of clinical settings, spanning regional anesthesia, minor surgical and dermatologic procedures, musculoskeletal injections, interventional pain techniques, and chronic or outpatient topical applications [1,2]. Despite this versatility, conventional formulations such as lidocaine, bupivacaine, and ropivacaine remain constrained by rapid systemic absorption, short intratissue residence time, and concentration-dependent toxicity [3]. These pharmacokinetic limitations often necessitate repeated injections, high-concentration dosing, or catheter-based continuous infusion. These strategies increase workflow complexity and may produce unpredictable systemic exposure or adverse effects [4,5].

Gel-based depots have emerged as a compelling approach for overcoming these constraints. By forming cohesive, space-filling matrices at the administration site, gels can sustain therapeutic local drug concentrations, attenuate systemic peaks, and shape release profiles that are more predictable or controllable than those of free drug injections [6]. Advances in polymer chemistry, supramolecular assembly, and stimuli-responsive design now enable gels that adapt to environmental cues such as temperature, pH, reactive oxygen species (ROS), or enzymatic activity, supporting context-sensitive LA delivery aligned with dynamic tissue conditions [7].

Within this broad class of depots, hydrogels and organogels represent two mechanistically distinct matrix architectures. Hydrogels are aqueous, polymer-based networks that form hydrated depots [8,9], whereas organogels rely on lipid or semi-polar continuous phases structured by supramolecular assembly of low-molecular-weight gelators (LMWGs) [10,11]. More recently, hybrid bigels integrating aqueous and lipid domains within a single biphasic matrix have been proposed as a conceptual extension of these systems [12,13]. Although these platforms are often discussed collectively as “gel-based depots,” their physicochemical properties, anatomical compatibility, and translational readiness differ markedly.

**Figure 1:**
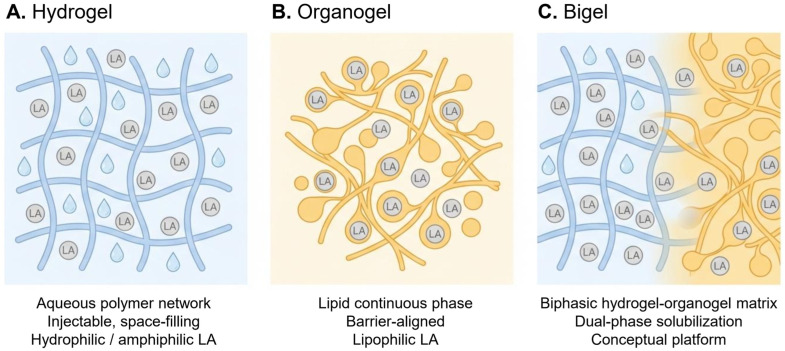
Conceptual architectures and drug compatibility of gel-based platforms for local anesthetic delivery. (**A**) Hydrogels consist of aqueous polymer networks that accommodate hydrophilic and amphiphilic local anesthetics and enable injectable, space-filling depots. (**B**) Organogels are structured by supramolecular assembly within lipid continuous phases, favoring solubilization and barrier-aligned delivery of lipophilic anesthetics. (**C**) Bigels integrate aqueous and lipid domains within a biphasic matrix, conceptually enabling dual-phase solubilization, although validated in vivo pharmacokinetic and analgesic data for local anesthetic delivery remain limited.

Given the expanding diversity of gel-based delivery technologies, there is a growing need for platform-level, decision-oriented synthesis that clarifies how matrix architecture governs drug solubilization, release behavior, barrier interaction, anatomical suitability, and translational readiness. Therefore, this review examines hydrogels, organogels, and bigels as distinct yet complementary classes of LA depots. Section 3 characterizes hydrogel architectures and their performance across nerve, wound, joint, and soft tissue models. Section 4 evaluates the supramolecular principles, barrier interactions, and translational evidence of organogels and emerging bigels. Section 5 provides an integrated mechanistic and translational comparison designed to guide platform selection in diverse clinical scenarios. Collectively, these sections offer a framework for advancing gel-based depots toward safer, longer-acting, and workflow-compatible LA delivery across a full spectrum of procedural, interventional, and outpatient contexts. To facilitate this platform-level comparison, the core structural architectures and drug compatibility characteristics of hydrogel-, organogel-, and bigel-based matrices are schematically summarized in Figure 1.

## 2. Methods: Literature Search and Study Selection

### 2.1. Literature Search Strategy

A structured literature search was conducted to identify preclinical and clinical studies evaluating gel-based platforms for LA delivery, with a specific focus on hydrogels, organogels, and emerging bigel systems. The following electronic databases were searched: PubMed/MEDLINE, Embase, and Web of Science. Studies published up to October 2025 were considered.

- Search terms were organized around three conceptual domains:
  (i)   LAs (e.g., lidocaine, bupivacaine, ropivacaine, tetracaine, prilocaine),
  (ii)  gel-based depots (e.g., hydrogel, thermogel, in situ gel, organogel, pluronic lecithin organogel, bigel, sustained release, stimuli-responsive), and
  (iii) delivery context or anatomical setting (e.g., perineural, nerve block, peri-incisional, infiltration, intra-articular, topical, transdermal, mucosal).
- A representative PubMed search query was as follows:
  (“hydrogel” OR “thermogel” OR “in situ gel” OR “organogel” OR “pluronic lecithin organogel” OR “bigel”) AND (“lidocaine” OR “bupivacaine” OR “ropivacaine” OR “tetracaine” OR “prilocaine” OR “local anesthetic”) AND (“delivery” OR “release” OR “perineural” OR “nerve block” OR “infiltration” OR “peri-incisional” OR “intra-articular” OR “topical” OR “transdermal” OR “mucosal”).

Reference lists of relevant articles and recent reviews were also screened to identify additional pertinent studies.

### 2.2. Eligibility Criteria

- Studies were considered eligible if they met the following criteria:
  (i)   the use of a defined gel-based matrix (hydrogel, organogel, or bigel),
  (ii)  inclusion of at least one LA payload, and
  (iii) reporting of release behavior, pharmacokinetics, analgesic outcomes, or biocompatibility in preclinical models or human subjects.

Preclinical studies were included across a range of anatomical and procedural contexts, including peripheral nerve blocks, peri-incisional or soft-tissue infiltration, intra-articular delivery, wound or implant-adjacent models, and dermal or mucosal applications. Clinical evidence encompassed approved topical or mucosal products, prospective clinical trials, and early-phase feasibility studies when the gel platform and anesthetic composition were clearly specified.

- Studies were excluded if they:
  (i)   did not include a LA payload (e.g., organogel studies using only non-anesthetic probes),
  (ii)  lacked a defined gel network structure (e.g., simple emulsions, ointments, or liquid formulations without depot behavior), or
  (iii) were not available in English.

Patent literature was not systematically searched; however, selected patents were cited when they explicitly described bigel-type architectures listing LAs as intended active agents.

### 2.3. Study Selection and Review Scope

Titles and abstracts were screened to exclude clearly irrelevant records, followed by full-text assessment of selected articles. Because this work is a narrative, platform-oriented review rather than a systematic review, a formal PRISMA workflow, exhaustive record counting, and quantitative risk-of-bias assessment were not performed.

Instead, studies were selected through an iterative, expert-curated process to ensure balanced representation across:

(i)   hydrogel architectures relevant to deep-tissue and injectable delivery,
(ii)  organogel systems aligned with dermal, transdermal, and mucosal barriers,
(iii) established and investigational clinical hydrogel products, and
(iv) emerging bigel concepts that inform hybrid matrix design, even when LA–specific in vivo data were limited.

To enhance transparency, the scope and type of evidence included for each platform category are summarized in Table 1, which outlines the nature of the preclinical and clinical evidence underpinning each major section of this review.

### 2.4. Review Focus and Framing

This review emphasizes platform-level comparison and decision-oriented synthesis, rather than exhaustive enumeration of all published formulations. The intent is to clarify how matrix architecture governs drug solubilization, release behavior, anatomical suitability, and translational readiness for LA delivery across procedural, interventional, and outpatient contexts. Accordingly, representative and mechanistically informative studies were prioritized over volume-based inclusion.

Throughout this review, the strength of evidence is discussed qualitatively according to study context rather than formal scoring systems. Preclinical evidence is primarily derived from rodent and large-animal models designed to assess depot retention, pharmacokinetics, and analgesic duration, whereas clinical evidence ranges from approved topical products and randomized trials to early-phase feasibility and non-inferiority studies. Accordingly, evidence is explicitly framed by level (preclinical vs. clinical), model relevance, and study design, with clear distinctions made between exploratory proof-of-concept studies, comparative preclinical experiments, and controlled human investigations. Because of substantial heterogeneity in models, comparators, and outcome measures, a formal risk-of-bias scoring framework was not applied; instead, critical appraisal is integrated narratively within each subsection.

## 3. Hydrogels for Local Anesthetic Delivery

Hydrogels are the most extensively investigated gel matrices for the controlled delivery of LA. Their three-dimensional, water-rich polymer networks form soft, tissue-compatible depots that sustain drug concentrations at the target site while reducing systemic exposure. Unlike particulate depots, such as microspheres or liposomes, hydrogels can be administered as cohesive, often injectable matrices that conform to irregular anatomical spaces [14].

The versatility of hydrogel design allows the modulation of crosslinking density, mechanical properties, and degradation behavior to achieve tailored release profiles [15]. Recent material innovations, ranging from thermoresponsive and covalently crosslinked architectures to pH-, ROS-, and enzyme-responsive systems, have expanded the functional landscape, enabling the co-delivery of anti-inflammatory, antimicrobial, or regenerative agents alongside LAs and supporting multimodal strategies that extend beyond strictly perioperative care [16,17].

This section summarizes the key material design principles, representative preclinical models across nerve, soft tissue, and joint environments, and the clinical and translational progress that collectively define the current state of hydrogel-based LA depots.

### 3.1. Material Design and Mechanistic Principles

Hydrogels for LA delivery rely on hydrated polymer networks that entrap, stabilize, and gradually release small molecules at therapeutically relevant levels. Key physicochemical parameters, including polymer composition, crosslink density, mesh size, water content, and degradation behavior, govern matrix rheology, drug mobility, and in vivo residence time [18]. Because these variables can be precisely tuned, hydrogels can be engineered to modulate depot stability, drug mobility, and in vivo residence time across a range of anatomical settings [19].

Advances in polymer chemistry have expanded this design space to include thermoresponsive, covalently crosslinked, stimuli-responsive, and multifunctional architectures capable of supporting both short-acting procedural anesthesia and sustained multimodal analgesia [20].

#### 3.1.1. Thermoresponsive Systems

Thermoresponsive hydrogels remain among the most clinically intuitive LA depots because their sol–gel transition aligns with standard procedural workflows. Poloxamer-based triblock copolymers exemplify this strategy; injected as low-viscosity liquids, they gel rapidly at body temperature to form cohesive depots that conform to irregular tissue planes and limit early drug efflux [21]. When loaded with bupivacaine or ropivacaine, these systems maintain local concentrations through micellization and polymer entanglement, while minimizing systemic absorption [22,23,24].

However, classical poloxamers exhibit low mechanical stiffness, dilution-induced erosion, and thermal storage instability [25]. Next-generation thermoresponsive systems, including poly(ethylene glycol)-poly(N-isopropylacrylamide)-poly(ethylene glycol) (PNDJ) hybrids and formulations incorporating poly(N-isopropylacrylamide), dextran derivatives, or polyester segments, address these limitations by increasing stiffness, prolonging residence time, and reducing burst release. By tuning the polymer hydrophobicity, block length, and chain architecture, these systems can be engineered with predictable gelation temperatures, viscoelasticity, and erosion profiles suited to environments ranging from peripheral nerve infiltration to joint spaces and mucosal cavities [26,27].

#### 3.1.2. Crosslinked and Dual-Network Architectures

To overcome the mechanical fragility of purely physically gelled systems, covalently crosslinked and dual-network hydrogels introduce secondary or permanent crosslinks that reinforce the polymer matrix and maintain depot integrity under mechanical strain. These materials resist deformation, swelling-driven disruption, and premature washout, features that are essential for deep soft tissue, perineural, intra-articular, and device-associated applications [28,29].

Hyaluronic acid (HA)–poloxamer hybrids illustrate this principle by combining the hydration and viscoelasticity of HA with the thermogelling behavior of poloxamers, yielding dual networks that resist early erosion while sustaining diffusion [30]. Enzyme-crosslinked gelatin–tyramine hydrogels formed via horseradish peroxidase–hydrogen peroxide coupling further demonstrate how tunable stiffness and biodegradable crosslinks can be integrated into injectable depots capable of withstanding a range of biomechanical forces [31]. Collectively, these platforms emphasize that mechanical robustness and controlled drug mobility must be co-optimized to achieve reproducible anesthetic performance in different tissue environments.

#### 3.1.3. Stimuli-Responsive Hydrogels

Stimuli-responsive hydrogels address a major limitation of conventional matrices, that is, their inability to adapt to changing biochemical environments. These “smart” systems synchronize LA release with local pathological cues through selective bond cleavage, matrix swelling, or accelerated degradation [32].

- pH-responsive hydrogels incorporate acid-labile linkages (acetals, hydrazones, imines) that cleave under mild acidosis found in inflamed or ischemic tissues [33].
- ROS-responsive networks rely on thioketal or boronic ester chemistry, which degrades in oxidative environments associated with early inflammatory bursts [34].
- Enzyme-responsive systems employ peptide crosslinkers cleavable by proteases, such as matrix metalloproteinase (MMP)-2 or MMP-9, which are upregulated during tissue remodeling, soft-tissue injury, or joint degeneration [35].

Across anatomical contexts, these platforms enable context-sensitive LA delivery that aligns drug exposure with evolving biological conditions while minimizing unnecessary dosing. Recent non-anesthetic studies further demonstrate that inflammation-responsive and self-healing injectable hydrogels can actively modulate oxidative stress, immune cell behavior, and tissue remodeling in complex inflammatory microenvironments, supporting the broader biological feasibility of such adaptive delivery systems [36].

Beyond endogenous biochemical cues, externally triggered stimuli-responsive hydrogels have been developed to enable user-controlled, on-demand anesthetic release. A representative example is a light-responsive macromolecular prodrug system in which tetracaine was covalently conjugated to poloxamer 407 via a photocleavable coumarin linker. In this platform, anesthetic activity remained suppressed in the absence of irradiation, while blue-light exposure triggered rapid cleavage and release of free tetracaine from an in situ–forming thermogel, producing adjustable and repeatable local nerve block in vivo. Although constrained by tissue penetration limits, such externally gated systems illustrate how hydrogel depots can decouple depot persistence from anesthetic exposure in superficial or cavity-accessible applications [37].

#### 3.1.4. Multifunctional and Composite Systems

Increasing emphasis has been placed on multifunctional hydrogels that deliver LAs together with agents that target inflammation, oxidative stress, infection, or early tissue repair. Co-delivery systems incorporating dexmedetomidine, non-steroidal anti-inflammatory drugs (NSAIDs), antioxidants, antimicrobials, or regenerative peptides support multimodal analgesia in both acute and chronic settings and broaden the therapeutic range of hydrogel depots [38,39]. Recent advances further illustrate how multifunctional hydrogel platforms can be engineered to actively modulate inflammatory and immune responses at tissue–material interfaces. For example, although not designed as an LA depot, a multifunctional hydrogel coating with ROS-scavenging activity demonstrated localized mitigation of oxidative stress, immunomodulation (macrophage polarization), and improved tissue integration in an implant-adjacent infection model, underscoring how next-generation hydrogels can function as biologically adaptive interfaces rather than passive depots [40].

Composite hydrogels incorporating nanoparticles, including liposomes, mesoporous silica, polymeric nanoparticles, and graphene-based fillers, introduce hierarchical diffusion barriers that generate biphasic or multiphasic release profiles suited to the early and late phases of tissue recovery [41,42]. Conductive, magneto-responsive, and piezoelectric fillers further expand the design space to include mechanoresponsive or electroresponsive hydrogels capable of on-demand or feedback-controlled analgesia [43,44].

#### 3.1.5. Mechanistic Continuum and Design Integration

Across these architectures, LA release follows a mechanistic continuum shaped by polymer chemistry, mesh structure, and degradation pathways. Early phase release is typically governed by Fickian diffusion through hydrated networks, whereas late-stage kinetics increasingly depend on hydrolysis, enzymatic cleavage, swelling, or erosion. The balance between diffusion- and degradation-driven release is dictated by the polymer concentration, hydrophilicity, degradable motifs, and crosslink density [15].

Rather than representing discrete categories, thermoresponsive, crosslinked, stimuli-responsive, and composite hydrogels occupy different regions within this design space, each emphasizing distinct strategies for controlling early drug retention, mechanical stability, or adaptive release [45]. Integrating these principles enables the rational engineering of hydrogel depots tailored to specific therapeutic objectives, ranging from short-duration procedural anesthesia to extended or context-sensitive analgesia in environments characterized by inflammation, oxidative stress, or mechanical loading. The key features of representative hydrogel design strategies, including their mechanistic drivers and preferred anatomical applications, are summarized in Table 2.

### 3.2. Preclinical Applications

Preclinical studies are central to defining the pharmacological rationale, safety profile, and mechanistic advantages of hydrogel-based LA delivery. Across neural, soft-tissue, musculoskeletal, and synovial models, hydrogels consistently retain drugs at the target site, sustain therapeutic tissue concentrations, and blunt systemic exposure relative to free-drug injections [46]. As each tissue environment presents distinct biochemical and mechanical constraints, these models collectively illustrate how hydrogel depots leverage prolonged release, structural stability, and tissue-adaptive behavior to overcome context-specific barriers. Representative findings are summarized across the three major experimental domains.

#### 3.2.1. Peripheral Nerve Block Models

Peripheral nerves exhibit rapid interstitial turnover and efficient systemic uptake, causing free LAs to dissipate within hours and produce high plasma maxima [47]. Thermoresponsive or self-assembling hydrogels can overcome these limitations by forming cohesive depots that conform to fascial planes and maintain favorable perineural diffusion gradients.

A Pluronic F-127/sodium hyaluronate hydrogel doubled the sensory block duration of bupivacaine and significantly prolonged motor blockade in beagle femoral and sciatic models without cardiotoxicity or functional impairment [48]. A dual-phase system combining HA–methacrylate microspheres with an oxidized-HA/carboxymethylcellulose matrix sustained ropivacaine release for 36–48 h in rats with no axonal injury [49]. A covalently crosslinked gelatin hydrogel (NHS–PEG–NHS) further demonstrated structural stability around neural tissues, reduced neurotoxicity in vitro, and prolonged sciatic blockade in mice [50].

Collectively, these results show that hydrogels enhance perineural localization, reduce early efflux, and extend therapeutic windows without compromising neural integrity, supporting their relevance in regional anesthesia models across species and nerve types.

#### 3.2.2. Surgical Wound and Soft-Tissue Infiltration Models

Soft-tissue environments, including incisional wounds, muscle planes, and mucosal surfaces, impose acidosis, enzyme activity, oxidative stress, and mechanical deformation, all of which hasten the clearance of conventional injections [51]. Despite these challenges, hydrogels, by forming mechanically resilient depots, preserve localization and prolong drug availability.

In rat incisional models, thermosensitive PLGA–PEG–PLGA depots extended ropivacaine-mediated analgesia to approximately 48 h without histologic irritation [52]. In a porcine peri-incisional model, a PNDJ hydrogel containing 4% bupivacaine reduced mechanical allodynia for up to 96 h and maintained at least seven days of tissue-level drug persistence, outperforming saline, liposomal bupivacaine, collagen sponge, polyorthoester, and bupivacaine HCl controls [53].

Composite designs provided additional benefits: a collagen–chitosan nanoparticle hydrogel co-loaded with bupivacaine and propolis accelerated wound closure and decreased interleukin-6 expression in rats [54], while a thermoresponsive polyelectrolyte gel delivering lidocaine enhanced mucosal healing and analgesia relative to several clinically used gels [55].

These models indicate that hydrogels stabilize depots against shear, exudate, and inflammatory turnover, improving analgesic durability while supporting tissue repair in both superficial and deep soft tissue environments.

#### 3.2.3. Intra-Articular Delivery Models

The joints exhibit rapid synovial dilution, short residence times, and sensitivity to LA-induced chondrotoxicity [56]. Thermoresponsive hydrogels counteract these constraints by forming viscoelastic depots that conform to joint biomechanics and resist washout.

A Pluronic F-127/HA hydrogel loaded with bupivacaine sustained analgesia for approximately 48 h in a rat osteoarthritis model, outperforming saline and blank gel [57]. A methylcellulose-based system modulated bupivacaine release according to pH and hyaluronan composition under synovial-mimicking conditions, demonstrating the feasibility of condition-adaptive joint dosing [58]. Reinforced hybrid systems combining poloxamer with self-assembling peptides increased the storage modulus, accelerated gelation, and prolonged intra-articular residence, with in vivo fluorescence detectable for up to four weeks [59]. In a rabbit knee surgery model, periarticular and intra-articular PNDJ hydrogels achieved 4–7 days of sustained bupivacaine exposure and significantly improved weight-bearing compared with liposomal bupivacaine [60].

These findings demonstrate that intra-articular hydrogels provide durable localization, adapt release to synovial biochemistry, and support functional recovery in load-bearing joints.

#### 3.2.4. Integrative Mechanistic Patterns

Across nerve, soft tissue, and joint environments, hydrogel depots exhibit consistent mechanistic behavior [18]:

- drug remains localized near the intended target,
- diffusion gradients favor sustained tissue-level exposure,
- systemic peaks are markedly attenuated, and
- degradation proceeds via hydrolytic, enzymatic, or redox-responsive pathways, without inducing fibrotic encapsulation.

Pharmacokinetic profiles typically exhibit plateau-like systemic curves rather than sharp maxima, reflecting controlled permeation through hydrated networks and gradual matrix breakdown [61]. These patterns collectively underpin the broad safety margin and spatial precision of hydrogel-based LA depots.

#### 3.2.5. Synthesis and Translational Implications

Across preclinical and early clinical studies, hydrogel-based LA depots consistently demonstrate the capacity to prolong local drug residence and extend analgesic duration relative to free-drug formulations. However, the translational significance of these findings depends less on peak analgesic efficacy than on reproducibility across anatomical sites, dosing conditions, and procedural workflows. Variability in injection volume, depot geometry, and surrounding tissue mechanics remains a major source of heterogeneity in reported outcomes [62].

From a translational perspective, future development will require standardized pharmacokinetic endpoints that capture local retention and systemic exposure in parallel, as well as harmonized in vitro–in vivo correlations to support rational dose selection. These considerations highlight that the primary barrier to broader clinical adoption is not proof of concept, but the need for controlled, procedure-specific validation frameworks that align hydrogel design with real-world anesthetic practice [18].

### 3.3. Clinical and Translational Progress

The clinical translation of hydrogel-based LA systems has advanced along two complementary pathways. One comprises well-established topical, dermal, and mucosal technologies that already provide predictable local anesthesia or analgesia with mature regulatory profiles, primarily in chronic or outpatient settings. The other consists of injectable and device-integrated depots that are beginning to demonstrate the feasibility of deeper-tissue, sustained-release analgesia in procedural contexts, including surgical, interventional, dental, dermatologic, and rehabilitative care. Collectively, these trajectories outline a maturing translational landscape in which surface-level hydrogel platforms have reached commercial stability, whereas deep-tissue depots remain under active investigation.

#### 3.3.1. Established Topical, Dermal, and Mucosal Systems

Topical lidocaine plasters are the most clinically mature hydrogel-based LA formulations. The 5% lidocaine medicated plaster (Lidoderm^®^; Endo Pharmaceuticals, Malvern, PA, USA/Versatis^®^; Grünenthal GmbH, Aachen, Germany) contains 700 mg of lidocaine in a hydrated adhesive matrix and achieves predictable dermal flux with systemic absorption of only approximately 3 ± 2% of the applied dose over 12 h, resulting in plasma concentrations < 0.3 µg/mL, well below toxicity thresholds [63,64]. Randomized trials in postherpetic neuralgia and focal neuropathic pain reported reductions in allodynia, improvements in daily pain scores, and decreased reliance on systemic analgesics, supporting guideline recommendations for first- or second-line use [65]. Sensory testing revealed minimal dense anesthesia beneath the patch, indicating that symptom relief primarily reflects modulation of ectopic nociceptor activity rather than full sensory blockade [66].

Hydrogel wound dressings extend this paradigm to painful acute and chronic wounds that require conformable moisture-compatible contact materials. The FDA-cleared Astero^®^ dressing (TRI-726 hydrogel containing 4% lidocaine; Gensco Pharma, Doral, FL, USA) provides rapid onset, multi-day pain reduction in venous ulcers, diabetic foot ulcers, pressure injuries, burns, and postsurgical incisions with no device-related complications [67,68]. Additional marketed matrices, including collagen–HA hydrogels with 2% lidocaine (Regenecare^®^ HA; MPM Medical, Inc., Mesquite, TX, USA) and silver–lidocaine composite films (MicroLyte^®^ Ag/Lidocaine; Imbed Biosciences, Inc., Fitchburg, WI, USA), couple anesthesia with antimicrobial or regenerative functions suitable for contaminated or chronic wound beds [69,70].

Mucosal hydrogels constitute another mature category. Oraqix^®^ (Dentsply Pharmaceutical, Inc., York, PA, USA), a poloxamer-based thermogelling formulation containing 2.5% lidocaine and 2.5% prilocaine, gels rapidly at intraoral temperatures and reduces procedural discomfort during scaling and root planing with minimal systemic absorption in multicenter trials [71]. Mucoadhesive films, such as Dentipatch^®^ (a 20% lidocaine transmucosal film; Noven Pharmaceuticals, Miami, FL, USA), provide extended transmucosal delivery for needle-site anesthesia in both adult and pediatric patients [72].

Collectively, these topical, dermal, and mucosal products demonstrate that hydrogel matrices are already embedded in routine clinical practice when superficial localized anesthesia or analgesia is required, primarily in chronic neuropathic or outpatient contexts, and establish a strong safety and pharmacokinetic foundation for the development of deeper-tissue depots.

#### 3.3.2. Injectable Thermogelling Depots

Early phase human studies have evaluated thermogelling hydrogels designed to transition from low-viscosity liquids to cohesive depots at body temperature. Among the most clinically advanced systems is PF72, a formulation based on poloxamer 407 with sodium hyaluronate. In randomized trials involving abdominal, maxillofacial, and esthetic procedures, PF72–ropivacaine infiltration reduced pain scores and analgesic requirements over 72 h compared with ropivacaine solution, degraded completely within approximately three days, and produced no device-related adverse events [73,74,75]. These findings highlight the feasibility of achieving controlled, short-term, deep-tissue analgesia without catheters or pumps.

The thermogelling feasibility has also been demonstrated with Welpass, a poloxamer–alginate hybrid. A single intra-procedural dose mixed with low-dose ropivacaine provided analgesia non-inferior to continuous catheter infusion in bariatric surgery patients while avoiding catheter-related logistical complexity, an advantage relevant across diverse patient populations, including those with a high body mass index or limited mobility [76].

These studies collectively support thermoresponsive hydrogels as promising candidates for controlled LA delivery in perioperative and interventional settings requiring precise localization and several hours to days of sustained effects.

#### 3.3.3. Non-Thermogelling and Viscous Biodegradable Depots

Parallel efforts have been made to evaluate non-thermogelling hydrogels and highly viscous biodegradable depots. Two randomized non-inferiority trials investigated a preformulated poloxamer 407 ropivacaine gel applied during thoracoscopic and laparoscopic procedures. A single intra-procedure application yielded 72 h analgesia comparable to continuous paravertebral or wound catheters, while avoiding catheter maintenance and demonstrating no impairment of wound healing [77,78].

In pediatric reconstructive surgery, a sodium-carboxymethylcellulose gel containing ropivacaine improved early donor-site pain control and reduced patient-controlled analgesia use relative to the free solution, illustrating the relevance of viscous depots in mechanically active tissues [79].

Established topical hydrogel platforms have also been repurposed for procedural applications. A triple-blind randomized trial of pre-incisional 5% lidocaine hydrogel plaster before craniotomy showed modest benefits in specific subgroups (such as male patients) and excellent tolerability [80]. Newer thin-film 1.8% lidocaine matrices exhibit strong adhesion and predictable delivery in phase 1 evaluation, suggesting deployment in humid or high-motion procedural environments [81].

These developments indicate that even in the absence of thermogelling behavior, preformed hydrogel matrices can function as durable depots across a range of procedural and non-procedural contexts.

#### 3.3.4. Implant-Integrated Constructs and Device-Assisted Depots

A growing translational direction involves the use of implant-integrated hydrogels and related device-assisted platforms. A gelatin-based bupivacaine-eluting ring co-implanted with pedicle screws provided multi-day localized release, substantially lower systemic peaks than infiltration, and favorable histological responses in a large animal spinal model [82]. Early human feasibility studies with related implant-linked depots indicated the potential for long-duration, anatomically anchored analgesia without reliance on external catheters or repeated injections [83].

Although mechanistically distinct from injectable depots, these implant-associated platforms broaden the landscape of gel-based analgesia, especially in orthopedic, spinal, dental, and device-intensive procedural fields.

#### 3.3.5. Translational Synthesis and Remaining Gaps

Although advanced and multifunctional hydrogel systems expand the conceptual design space for LA delivery, their increasing complexity introduces new challenges that may constrain translational feasibility. The incorporation of responsive linkers, secondary payloads, or composite fillers complicates manufacturing control, sterilization, and batch-to-batch reproducibility. In addition, context-sensitive release mechanisms depend on biological triggers that may vary substantially between patients and disease states, raising concerns regarding under- or over-release in clinical settings.

These limitations underscore the importance of aligning hydrogel complexity with a clearly defined clinical objective. For applications requiring predictable and robust analgesia, simpler architectures with well-characterized diffusion and degradation profiles may offer superior translational reliability. More complex, adaptive systems may be better suited to exploratory or high-risk indications, but will require rigorous validation to establish safety margins and controllability before clinical deployment.

From a clinical-context perspective, the current evidence is bimodal: surface-level products are primarily used in chronic neuropathic or wound-related pain, whereas injectable and implant-integrated depots target perioperative and interventional analgesia. Making this distinction explicit is essential when comparing hydrogels with organogels and bigels as LA platforms. Representative clinical hydrogel-based LA platforms, including established topical products and investigational injectable or implant-integrated depots, are summarized in Table 3.

### 3.4. Challenges and Future Outlook

Despite substantial progress, hydrogel-based LA systems continue to face material, manufacturing, regulatory, and clinical deployment challenges that limit their broader adoption. As these depots are placed in anatomically sensitive or mechanically active environments, including perineural spaces, synovial cavities, musculoskeletal interfaces, donor sites, dermatologic surfaces, and implant-adjacent tissues, even small deviations in exposure can lead to neurotoxicity, chondrotoxicity, and inadequate analgesia [84]. Consequently, hydrogel depots operate within a relatively narrow therapeutic window and must simultaneously achieve mechanical stability, predictable degradation, regulatory reproducibility, and seamless integration into diverse procedural and outpatient workflows.

#### 3.4.1. Material and Formulation Constraints

A central material challenge is the coordinated optimization of mechanical robustness, controlled biodegradation, and spatiotemporally precise drug release. Classical thermoresponsive and physically crosslinked gels are prone to dilution-induced erosion, mechanical deformation, and burst release when loaded with high LA concentrations [61]. Increasing crosslink density or introducing dual networks can reinforce structural stability, but often tightens mesh size, slows diffusion, and complicates complete resorption [85].

These trade-offs are particularly relevant for LAs, many of which are administered near their maximum safe concentrations. Overly dense networks may delay onset by retaining the drug, whereas under-crosslinked matrices can permit rapid efflux and systemic peaks that narrow the safety margin. High LA loading can also disrupt polymer self-assembly, induce phase separation or crystallization, and unpredictably shift the release kinetics [8,49]. Therefore, achieving both structural integrity and reliable pharmacokinetics in a single depot remains a key design challenge for both perioperative and chronic applications.

#### 3.4.2. Challenges Unique to Stimuli-Responsive Systems

Stimuli-responsive hydrogels introduce additional complexities. pH-, ROS-, and enzyme-responsive linkages aim to synchronize release with local biochemical conditions, such as acidosis, oxidative stress, or protease activity, but these cues vary substantially across individuals, interventions, and healing trajectories [33,34,35]. Insufficient acidosis or ROS generation may result in under-release and incomplete analgesia, whereas excessive inflammation may accelerate degradation and shorten depot persistence [86].

Multi-stimuli systems, nanoparticle-reinforced matrices, and electroresponsive or mechanoresponsive composites require precise control of interfacial chemistry to avoid batch-to-batch variation or unanticipated local reactions [14,87]. Designing responsive systems that maintain robustness despite biological heterogeneity while offering clear advantages over simpler non-responsive depots remains an unresolved materials-science challenge.

#### 3.4.3. Manufacturing, Sterilization, and Regulatory Barriers

Most hydrogel–LA depots qualify as combination products, requiring independent verification of the active drug, polymer matrix, and any associated delivery components or implantable elements [88]. Scaling benchtop formulations into clinical-grade products demands consistent control over the polymer molecular-weight distribution, degree of functionalization of degradable or responsive linkers, and residual monomer or crosslinker content [89].

Sterilization is a major bottleneck; gamma irradiation and autoclaving may alter gelation temperature, crosslink density, or rheology, whereas aseptic processing and low-temperature methods increase manufacturing complexity and cost [90,91,92]. Stability studies must ensure that storage does not alter the phase behavior or compromise the responsiveness. Compounding these challenges is the absence of widely accepted in vitro–in vivo correlations, which forces reliance on iterative animal testing and small early phase human studies [93]. Heterogeneity in release-testing conditions, pharmacokinetic sampling protocols, and clinical endpoints further complicates regulatory evaluation [18].

#### 3.4.4. Practical Barriers in Clinical Deployment

Successful deployment depends on procedural variables such as volume, concentration, injectate viscosity, and placement plane, all of which strongly influence depot performance [94]. Depots must be deliverable through fine-gauge needles or blunt cannulas without excessive force, and must gel or set rapidly enough to prevent intravascular spread or uncontrolled dispersion. Perineural applications can be affected by fascial irregularities, fibrosis, or operator variability; intra-articular and musculoskeletal sites must withstand mechanical loading and tissue strain, and mucosal or dermal surfaces introduce their own hydration and turnover constraints [95].

Workflow integration also varies across institutions; anesthesiologists, surgeons, emergency physicians, dentists, dermatologists, and interventional specialists may all place hydrogel depots, each with different timing preferences, procedural exposure, and visualization methods [96]. Comparator arms in clinical trials, including single-shot injections, catheter-based infusions, systemic analgesics, and multimodal regimens, make it difficult to determine where hydrogel depots confer the greatest incremental benefit [97,98]. Methods to verify depot localization and persistence (ultrasound, magnetic resonance imaging, fluorescent tracers, or surrogate pharmacokinetic markers) remain exploratory and lack a standardized interpretation [99].

#### 3.4.5. Health-System Constraints and Evidence Requirements

For broad adoption, hydrogel depots must demonstrate not only analgesic benefits, but also operational and economic feasibility. Cost-effectiveness relative to catheters, systemic regimens, and device-based alternatives is essential. Additional procedural time must be minimal, and failure modes should be predictable and manageable with standard analgesics [100]. Large multicenter randomized trials and pragmatic real-world studies are required to validate safety, usability, and performance across diverse patient populations and procedural contexts. Without such evidence, integration into clinical pathways and reimbursement frameworks remains limited.

#### 3.4.6. Future Directions in Materials, Computation, and Smart Delivery

Several converging technological advances have offered a clear forward trajectory. Innovations in degradable linkers, micro- and nanostructured composites, and bio-orthogonal chemistries enable hydrogels to be mechanically resilient yet fully resorbable, with degradation kinetics aligned to the time course of acute or subacute pain [101,102]. Hierarchical designs that embed nanoparticles, liposomes, or micelles can support early- and late-phase release profiles aligned with activity levels or tissue healing [103,104]. Multi-payload depots combining LAs with α_2_-agonists, NSAIDs, corticosteroids, biologics, or regenerative cues may integrate analgesia with inflammation control or early tissue repair [23,105].

Machine-learning-assisted formulation tools and pharmacokinetic-pharmacodynamic digital twins may streamline optimization and support individualized dosing strategies [106,107,108,109]. Advances in low-temperature sterilization and real-time release testing can improve the lot-to-lot consistency [91,92]. Ultimately, the most transformative direction lies in feedback-responsive hydrogels capable of adjusting release in response to local pH, ROS, enzymatic activity, mechanical strain, or even neural signaling [7,110]. Although largely preclinical, these platforms represent credible pathways for biologically synchronized analgesia.

Taken together, these developments position hydrogels as the most advanced polymeric platforms for deep-tissue, space-filling LA depots, while maintaining a strong foothold in chronic and outpatient surface-level applications. At the same time, lipid-rich organogels and emerging bigel hybrids occupy distinct niches within the broader universe of gel-based delivery technologies. Their comparative features, limitations, and translational implications are examined in Section 4, which sets the stage for an integrated platform-level perspective in the concluding section.

## 4. Organogels for Local Anesthetic Delivery

Organogels represent a non-aqueous class of gel matrices formed by the supramolecular assembly of LMWGs in organic or semi-polar solvents, producing semi-solid viscoelastic depots with a strong affinity for lipophilic drugs. Their hydrophobic continuous phase markedly improves solubilization and retention of lipid-soluble LAs, such as bupivacaine, ropivacaine, and tetracaine, positioning organogels as promising vehicles for topical, transdermal, and mucosal anesthesia, where gradual tissue penetration and localized action are desired [10,11].

This physicochemical profile aligns organogels with topical, transdermal, and mucosal anesthesia, where gradual tissue penetration and localized action are favored. By varying gelator chemistry, solvent composition, and excipient content, organogel properties such as firmness, spreadability, and depot persistence can be tuned without reliance on aqueous polymer backbones [111,112].

This section reviews the molecular and supramolecular principles governing organogel assembly, summarizes representative preclinical evidence in dermal, transdermal, and mucosal models, and examines available clinical experience, together with translational challenges and emerging hybrid bigel strategies that may extend the capabilities of traditional organogel depots.

### 4.1. Molecular Assembly and Network Chemistry

The organogel performance in LA delivery is fundamentally dictated by the molecular architecture of the gel network and supramolecular interactions that stabilize the non-aqueous matrix. Unlike hydrogels, which rely on water-swollen polymer meshes, organogels are built from the self-assembly of LMWGs in organic or semi-polar solvents [113]. Gelator chemistry, solvent polarity, microstructural organization, and excipient composition collectively influence network formation, thereby determining drug solubilization, depot stability, and release kinetics in surface-directed anesthetic applications [114].

#### 4.1.1. Supramolecular Assembly and Representative Gelator Families

Organogel formation is driven by the supramolecular assembly of LMWGs into viscoelastic networks that immobilize the surrounding solvent without requiring covalent crosslinking [115]. These assemblies rely on reversible non-covalent interactions, including hydrogen bonding, π–π stacking, van der Waals forces, solvophobic aggregation, and ion pairing in phospholipid systems [116]. Through these interactions, gelators organize into fibrillar, lamellar, or particulate microstructures that entrap the solvent phase and modulate the mobility of the embedded LAs [117,118,119].

Among LMWGs, lecithin-based systems are the most extensively characterized for anesthetic delivery. Lecithin self-assembles in non-polar solvents, such as isopropyl myristate or medium-chain triglycerides, to form elongated reverse micelles that interconnect into worm-like networks with shear-thinning behavior, thermal reversibility, and tunable viscoelasticity [120]. These lipid-rich microdomains solubilize lipophilic anesthetics, including tetracaine, benzocaine, lidocaine, and ropivacaine, while limiting crystallization and enhancing depot stability [121].

Other gelators, such as sorbitan esters, fatty acids, monoglycerides, cholesteryl derivatives, and amino acid–based gelators, form organogels through hydrophobic interactions, hydrogen-bonded fibrils, or π-stacked fibers. These mechanistic differences influence the solubilization efficiency, barrier penetration, and sustained-release behavior in LA depots [122,123,124].

#### 4.1.2. Solvent and Co-Excipient Modulation of Network Structure and Drug Behavior

The organic continuous phase strongly influences both network morphology and anesthetic behavior. Solvent polarity, viscosity, and lipophilicity determine gelator packing and modulate LA solubilization and partitioning [125]. Medium-chain triglycerides, oleic acid, propylene glycol dicaprylocaprate, and isopropyl palmitate each generate distinct microstructures that affect the firmness, occlusiveness, and diffusion pathways [126,127]. Highly lipophilic solvents favor deep incorporation of bupivacaine or ropivacaine within the continuous phase, whereas less hydrophobic solvents promote LA association with gelator fibrils or interfacial domains, thereby altering diffusion resistance and release kinetics [125,127].

Co-excipients provide additional levers for modulating the organogel architecture. Small amounts of water, short-chain alcohols, fatty acids, or surfactants can plasticize the network, modify gelator packing, or generate bicontinuous microdomains that alter LA mobility. For instance, trace hydration in lecithin organogels induces transitions from reverse micelles to lamellar or bicontinuous structures, increasing fluidity and shifting diffusion profiles [128]. Permeation enhancers, such as oleic acid or terpenes, may simultaneously alter stratum corneum lipids and interact with the organogel matrix to enhance partitioning and release [10,129].

#### 4.1.3. Mechanistic Implications for Local Anesthetic Delivery

Together, gelator assembly, solvent–gelator interactions, microstructural transitions, and excipient-driven modulation determine the mechanical stability and release behavior of organogels used for LA delivery. Their reversible, non-covalent architecture creates a tunable depot in which modest compositional adjustments can produce substantial changes in persistence, drug availability, and tissue penetration [121,130]. These mechanistic distinctions from aqueous hydrogels underpin the utility of organogels as surface-directed anesthetic depots and explain their growing relevance in topical, transdermal, and mucosal delivery contexts, particularly in outpatient or chronic pain scenarios.

### 4.2. Preclinical Applications

Preclinical evaluations of organogel-based LA delivery are fewer in number than the corresponding hydrogel investigations, yet they collectively demonstrate a coherent pharmacological rationale. Most studies have focused on dermal and transdermal routes, with a smaller but growing set examining oral and other mucosal applications that present higher moisture, enzymatic turnover, and mechanical stress. Across these settings, organogels enhance the solubilization of lipophilic LAs, modulate permeation across barrier tissues, and prolong local residence with acceptable cytocompatibility. Complementary ex vivo transport studies provide mechanistic clarity on how organogel microstructure, lipid composition, and polymer–surfactant interactions govern drug flux and tissue distribution.

#### 4.2.1. Dermal and Transdermal Skin Models

Dermal and transdermal delivery remain the most extensively studied domains of organogel-based LA depots. Nanostructured lipid–poloxamer organogels formulated with oleic acid, lanolin, and poloxamer 407 produce dense viscoelastic matrices that efficiently solubilize lidocaine and maintain structural cohesion on the skin surface. Increasing lanolin and poloxamer content modulated phase-transition behavior and produced elastic–viscous modulus ratios near 15, indicating enhanced mechanical stability. Ex vivo porcine skin testing showed that optimized formulations flattened release curves, reduced lidocaine flux from approximately 17 to 12 μg/cm^2^/h, and decreased permeability coefficients from 1.2 to 0.62 per hour. In vivo tail-flick assays confirmed prolonged dermal anesthesia relative to commercial gels and creams, despite containing only 2% lidocaine, while keratinocyte cytotoxicity remained within acceptable limits [131].

A related formulation strategy incorporated lidocaine hydrochloride and a synthetic monoketonic curcuminoid into hybrid poloxamer–lipid organogels composed of a Pluronic F127 aqueous phase and isopropyl myristate–lecithin organic phase. Lecithin and curcuminoid addition enhanced viscoelasticity, altered sol–gel transition behavior, and produced sustained lidocaine release across Strat-M membranes, with flux increasing from roughly fifteen to twenty micrograms per square centimeter per hour compared to lecithin-free controls. Although behavioral testing was not performed, rheological and permeation findings indicate that tuning the poloxamer–lipid composition can synchronize the depot structure with controlled anesthetic release [132].

Collectively, these dermal studies demonstrate that lipid-rich, poloxamer-structured organogels achieve high lidocaine solubilization, controlled rheology, and sustained cutaneous exposure compared to conventional liquid or cream vehicles, while limiting systemic uptake. From a clinical framing perspective, these models primarily simulate topical or transdermal outpatient use rather than acute peri-incisional analgesia.

#### 4.2.2. Mucosal and Cavity Models

Mucosal and cavity environments pose greater challenges owing to constant moisture, enzymatic activity, and mechanical disturbance. Thermoreversible mucoadhesive organogels formulated from a PEG–poloxamer aqueous phase, PEG 4000, and Sacha inchi oil exhibit low-temperature injectability with rapid gelation near physiological conditions. These systems exhibit increased viscosity and strong mucoadhesion, allowing stable retention in the oral cavity geometries. In artificial saliva, they sustained lidocaine release for approximately 72 h, while in vivo tail-flick and hot-plate assays demonstrated significantly prolonged analgesia compared with lidocaine hydrogel or aqueous solution, indicating superior resistance to salivary washout and enhanced depot persistence in moisture-rich environments [133].

In addition to oral models, additional mucosal applications have been explored; however, comprehensive datasets remain scarce. The dry-socket framework represents the most complete characterization to date and underscores the need for broader evaluation of gynecologic, anorectal, and ocular anesthesia, where organogels could support procedural or post-procedural analgesia via cavity-accessible routes.

#### 4.2.3. Mechanistic Barrier Insights and Translational Implications

Although many organogel studies use non-anesthetic probes, these experiments provide essential mechanistic insights into barrier transport, microstructural behavior, and depot integrity. Pluronic–lecithin organogels (PLOs), frequently evaluated as transdermal vehicles, consistently demonstrate enhanced skin retention and, in some cases, increased systemic uptake relative to cream bases [134]. These effects are attributed to lecithin- and isopropyl palmitate-induced fluidization of stratum corneum lipids together with poloxamer-mediated stabilization of hydrophobic solubilizing domains [132].

Lanolin-derived organogels developed for other lipophilic drugs further illustrate how oil type, gelator concentration, and permeation enhancers modulate diffusion flux, lag time, and partitioning in mammalian skin [135]. While these studies did not measure analgesic outcomes, they revealed physicochemical determinants governing whether a lidocaine-loaded organogel behaves primarily as a high-flux penetration enhancer or as a depot emphasizing localized retention.

Taken together with LA-specific dermal and mucosal models, these findings support a dual mechanistic rationale for organogel-based LA delivery: enhanced solubilization and stabilization of lipophilic anesthetics within structured lipid–surfactant matrices and tunable modulation of barrier properties that balance penetration depth with local retention. Dermal studies demonstrated prolonged lidocaine action with attenuated systemic exposure and limited cytotoxicity [131], whereas mucosal models showed sustained analgesia in moisture-rich, mechanically disruptive environments [133].

At the same time, the evidence base is narrow. Lidocaine dominates as the test anesthetic, dermal models predominate, and robust in vivo comparisons involving ropivacaine, bupivacaine, and tetracaine are rare. Many mechanistic insights have been extrapolated from ex vivo barrier studies or non-anesthetic probes, and few models directly capture surgical incisions or other acute perioperative settings. Systematic in vivo evaluation in standardized incisional pain models, along with comparative pharmacokinetic analyses against established topical systems, remains an unmet need.

Within the broader context of this review, current organogel data therefore align more closely with chronic neuropathic, focal musculoskeletal, and outpatient topical use, while only indirectly informing potential perioperative roles, such as pre-procedural skin or mucosal anesthesia. To consolidate these preclinical findings and contextualize them alongside the available clinical evidence and emerging bigel concepts, the key organogel- and bigel-based LA delivery platforms are summarized in Table 4.

### 4.3. Clinical Experience

Although organogels have been extensively evaluated as topical and transdermal carriers for various therapeutics, clinical studies specifically examining LA-loaded organogels remain limited. Most available human data originate from compounded formulations used in chronic pain practice rather than standardized or commercially developed products. The following examples summarize the best-characterized clinical applications to date and define the current scope of evidence.

#### 4.3.1. Structured Human Evaluation of PLO-Based Lidocaine Organogels

The most rigorously conducted study of an organogel-based LA formulation is a randomized, double-blind, placebo-controlled crossover trial investigating the incorporation of 5% lidocaine into PLO. The organogel was produced by combining a lecithin–isopropyl myristate organic phase with a poloxamer aqueous phase, yielding an amphiphilic matrix capable of solubilizing lipophilic drugs. Patients with postsurgical neuropathic pain, postherpetic neuralgia, or diabetic neuropathy applied the preparation twice daily for one-week treatment periods.

Lidocaine–PLO produced a modest but statistically significant reduction in pain intensity relative to the baseline, whereas an amitriptyline–PLO formulation showed no meaningful improvement. However, lidocaine and placebo achieved similar overall reductions, and the magnitude of benefit with lidocaine fell below the clinically important thresholds. Local tolerability was favorable, with itching being the most common adverse event, and no systemic toxicity observed. This trial, therefore, demonstrates that lidocaine can be stably incorporated into a PLO matrix and used safely in human subjects, but it also highlights the limited incremental efficacy over placebo in chronic neuropathic indications [136].

#### 4.3.2. Compounded PLO Organogels in Clinical Practice

Beyond controlled trials, PLO organogels have been widely used as compounded preparations for chronic pain management. These formulations often combine lidocaine with other agents, such as ketamine, baclofen, or amitriptyline, and are used for refractory neuropathic or focal musculoskeletal pain. Evidence supporting these applications consists largely of retrospective series, case reports, and clinical practice summaries [134].

Although these reports collectively suggest that organogels can serve as practical topical delivery vehicles for LAs, they typically lack standardized formulations, prospective controls, or pharmacokinetic characterization [139]. Consequently, the contribution of the organogel matrix, versus the active drug or adjunctive agents, to the observed clinical effects remains difficult to determine.

#### 4.3.3. Overall Clinical Evidence Landscape

Current human experience demonstrates the feasibility of using lipid-rich amphiphilic organogels, such as PLO, for topical LA delivery. However, the clinical evidence remains narrow. Existing studies have focused primarily on chronic neuropathic pain rather than procedural or perioperative analgesia, and comparative data against established topical anesthetic systems, such as lidocaine plasters or hydrogel matrices, are scarce.

Thus, the available literature reflects early clinical exploration rather than mature validation. More systematic studies are needed to assess formulation reproducibility, local and systemic pharmacokinetics, and therapeutic performance across dermatologic, dental, and minor procedural settings. In the context of this review’s broader scope, current clinical data position organogel-based LAs mainly within the chronic and outpatient topical and neuropathic domains, with perioperative applications still speculative and largely unsupported by trial-level evidence.

### 4.4. Challenges and Future Directions

Despite the clear mechanistic advantages of delivering lipophilic LAs, organogel technologies face several scientific, translational, and regulatory barriers that limit their broader clinical deployment. As development shifts from empirically compounded formulations to rationally engineered systems, three domains remain central to future progress: physicochemical and mechanistic constraints inherent to non-aqueous matrices; translational and regulatory limitations arising from formulation heterogeneity and limited clinical data; and emerging opportunities to integrate aqueous and non-aqueous networks through hybrid bigel architectures.

#### 4.4.1. Physicochemical and Mechanistic Challenges

The hydrophobic continuous phase of organogels is well suited for solubilizing lipophilic anesthetics, yet it also imposes several constraints. Reversible self-assembly of LMWGs introduces temperature-sensitive stability and batch variability, complicating the precise control of viscoelasticity and diffusion pathways. The accommodation of hydrophilic anesthetic salts, such as lidocaine HCl, is limited and often requires ion pairing, co-solvents, or hybrid formulations to achieve reliable solubilization.

Barrier modulation is an additional challenge. Lecithin and isopropyl palmitate can enhance partitioning into the stratum corneum but may transiently weaken barrier integrity, raising concerns regarding long-term or repeated application. Finally, organogels function primarily as surface-directed depots, restricting their relevance to dermal, mucosal, or cavity-accessible tissues, and limiting their current applicability to deeper procedural or perioperative analgesia [140,141].

#### 4.4.2. Translational, Clinical, and Regulatory Limitations

In contrast to hydrogels, several of which have achieved regulatory approval, organogel-based anesthetic systems remain largely confined to compounded clinical practice. Variability in gelator grade, lecithin purity, oil identity, hydration level, and permeation enhancers results in wide differences in rheology, microstructure, and release kinetics [10,121,132]. This heterogeneity undermines reproducibility and impedes the establishment of standardized safety and pharmacokinetic profiles.

Human studies are limited to small trials and observational reports, predominantly on chronic neuropathic pain. Comprehensive pharmacokinetic analyses quantifying local tissue concentrations, depot depletion dynamics, and systemic absorption are lacking. Comparative studies against established topical anesthetic formulations such as lidocaine plasters or hydrogel matrices are sparse. These gaps present significant challenges to formal regulatory evaluation and hinder wider adoption, particularly in perioperative environments where highly predictable exposure profiles are required.

#### 4.4.3. Emerging Bigel Platforms as Hybrid Strategies

Bigels, which are biphasic systems consisting of interpenetrating hydrogel and organogel domains, have gained attention as next-generation platforms capable of overcoming several limitations of classical organogels. Structurally, bigels combine an organogel domain that solubilizes and stabilizes lipophilic LAs (for example, tetracaine, bupivacaine, or ropivacaine) with a hydrophilic hydrogel component that enhances mucoadhesion, modulates mechanical properties, accommodates hydrophilic co-solutes, and enables additional release-control mechanisms [12,142].

Several mechanistic advantages hold particular relevance for LA delivery.

- Dual-domain partitioning: Lipophilic anesthetics preferentially reside in the organogel phase, while hydrophilic agents or buffers localize within the hydrogel domain, reducing crystallization and enabling multi-agent strategies [143].
- Improved spreadability: Hydrogel incorporation reduces greasiness and promotes uniform topical deposition, addressing the key usability limitations of classical organogels [144].
- Enhanced mechanical stability: Interpenetrated polymeric networks reduce the temperature and shear sensitivity of LMWG-based gels, improving rheological consistency across environmental conditions [145].
- Modulated release kinetics: Biphasic release patterns, with initial hydrogel-mediated diffusion followed by slower organogel-controlled efflux, may support smoother, prolonged anesthetic exposure [12].

Although bigels have not yet been directly evaluated for LA delivery in peer-reviewed in vivo studies, multiple lines of indirect evidence support their translational potential. Bigels formulated with ibuprofen, metronidazole, and other locally active agents demonstrate enhanced rheology, increased dermal deposition, and more controlled release than single-phase gels [137,138]. In parallel, extensive literature has demonstrated that LAs can be effectively retained and released from both lipid-based organogels and polymeric hydrogels individually [121,146], providing a mechanistic foundation for anticipating similar behaviors in hybrid systems.

Patent literature further strengthens this conceptual link, describing bigel compositions that explicitly list lidocaine and related LAs as suitable active ingredients for topical or transdermal applications [147]. Collectively, these findings position bigels as a rational next step toward more robust and tunable surface-directed anesthetic depots, with the potential to support both outpatient chronic use and selected peri-procedural topical applications once in vivo LA data emerge.

#### 4.4.4. Directions for Future Research

Advancing organogels and bigels toward clinical translation will require progress in several areas [88,148,149]:

1. Standardized formulation frameworks: Establish reproducible gelator–solvent ratios, hydration levels, and rheological benchmarks to minimize variability and support multicenter comparisons.
2. Mechanistic pharmacokinetics: Quantify tissue partitioning, depot depletion, and systemic absorption across dermal, mucosal, and cavity models, ideally including head-to-head comparisons with hydrogels, creams, and patches.
3. Comprehensive safety profiling: Assess long-term tolerability, barrier integrity, and excipient biocompatibility, particularly for formulations containing permeation enhancers or high lecithin content.
4. Expansion into procedural models: Evaluate performance in surgical incision, cavity packing, wound-edge infiltration, and minimally invasive procedural anesthesia to clarify whether organogel or bigel depots can add value in perioperative settings.
5. Systematic bigel development: Optimize hydrogel–organogel ratios, interfacial stabilization, and multi-payload loading strategies to leverage the full potential of biphasic architectures for both chronic and procedural use.

#### 4.4.5. Integrative Perspective

Organogels and emerging bigel platforms constitute a distinct, surface-oriented class of LA depots, characterized by a strong affinity for lipophilic agents and highly customizable supramolecular architectures. Their physicochemical behavior, barrier interactions, and translational constraints differ fundamentally from those of aqueous hydrogel systems.

In clinical terms, current evidence anchors organogels primarily in chronic neuropathic and outpatient topical or wound-related analgesia, with perioperative or procedural applications still at the level of conceptual or early preclinical exploration. Bigels offer a plausible route toward more robust and tunable barrier-aligned depots but remain at a formulation and non-LA proof-of-concept stage.

A platform-level comparison of these modalities, including mechanistic features, perioperative versus chronic application niches, and regulatory maturity, is provided in Section 5 and is essential for positioning hydrogels, organogels, and bigels within the broader landscape of gel-based LA delivery.

## 5. Platform-Level Comparison and Decision Framework

Gel-based LA depots operate within fundamentally different matrix architectures that shape solubilization, retention, release behavior, anatomical suitability, and translational feasibility. Hydrogels, organogels, and bigels are not competing alternatives in a linear hierarchy; rather, each occupies a distinct position within a multidimensional design space defined by (1) continuous-phase chemistry, (2) drug–matrix compatibility, (3) interaction with tissue-specific barriers and mechanical forces, and (4) regulatory and manufacturing constraints that govern real-world deployability.

This section integrates the mechanistic, experimental, and translational evidence from Section 3 and Section 4 into a decision-oriented framework that clarifies how these platforms should be matched to clinical scenarios across the full spectrum of topical, infiltrative, interventional, and chronic pain applications.

### 5.1. Architectural Axes That Determine Platform Behavior

Across gel depots, three architectural axes dominate LA handling and clinical performance [8,12,143,150,151,152,153].

#### 5.1.1. Axis 1: Continuous-Phase Architecture

- Hydrogels feature an aqueous continuous phase supported by crosslinked or physically associated polymer networks. This structure provides hydrophilic diffusion pathways, isotropic swelling, and compatibility with hydrophilic and amphiphilic anesthetics.
- Organogels use lipid or semi-polar solvents structured by LMWGs, creating hydrophobic continuous phases with a high affinity for lipophilic LA bases and facilitating efficient barrier partitioning across lipid-rich interfaces.
- Bigels integrate both aqueous (hydrogel) and lipid (organogel) domains as interpenetrating or co-continuous phases, enabling dual solubilization and modulating the rheology and diffusion behavior of the parent organogel.

#### 5.1.2. Axis 2: Drug-Matrix Compatibility

Drug ionization and lipophilicity strongly influence depot selection:

- Hydrogels efficiently retain ionized or amphiphilic LAs via mesh entrapment or responsive linkages.
- Organogels excel in solubilizing neutral or lipophilic LA bases, which readily partition into lipid matrices.
- Bigels offer a conceptual compromise, allowing lipophilic drug loading in lipid domains while incorporating hydrophilic excipients or buffers in aqueous regions.

#### 5.1.3. Axis 3: Structural and Biomechanical Stability

- Hydrogels demonstrate conformability, injectability, and mechanical resilience in dynamic or hydrated planes, such as perineural, peri-incisional, intra-articular, or implant-adjacent spaces.
- Organogels maintain semi-solid cohesion on the skin or mucosa but lose stability in high-moisture or high-motion environments.
- Bigels improve upon the mechanical weaknesses of organogels through aqueous-phase reinforcement, although their deep-tissue stability remains untested.

Taken together, these three axes predict where each platform is best suited anatomically and clinically, forming the basis for the scenario-specific mapping described in Section 5.2.

### 5.2. Anatomical and Clinical Scenario Mapping

**Figure 2:**
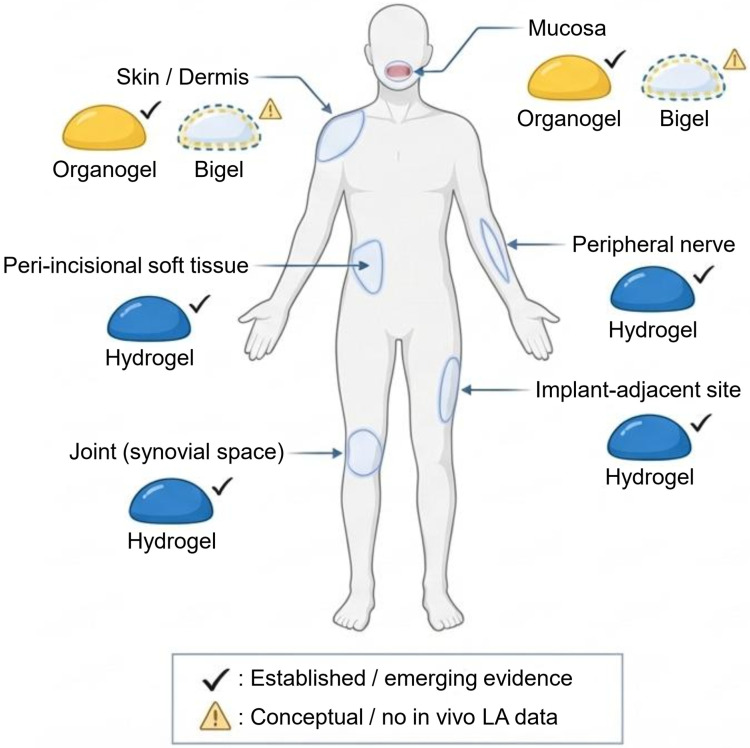
Platform–anatomy matching and ideal clinical microenvironments for gel-based local anesthetic depots. Hydrogels demonstrate the broadest anatomical compatibility, including perineural, peri-incisional, intra-articular, and implant-adjacent settings, reflecting their injectability and mechanical stability in hydrated or dynamic tissues. Organogels are primarily aligned with dermal, transdermal, and mucosal applications, where lipid-rich matrices support barrier-oriented delivery. Bigels remain conceptual platforms, proposed mainly for surface or interface applications, with limited translational and in vivo validation. Differences in evidence maturity and translational readiness across platforms are highlighted.

Matrix–tissue compatibility depends on barrier composition, hydration, motion, geometry, and required depot persistence. When functionalized through the architectural axes above, each platform aligns with distinct clinical niches [10,13,112,129,154,155,156,157]. Based on the architectural axes outlined above, the alignment of each gel platform with ideal anatomical microenvironments and clinical scenarios is schematically summarized in Figure 2.

#### 5.2.1. Skin, Dermal, and Transdermal Applications

- Organogels (ideal): lipid continuity matches the stratum corneum, enabling high partitioning of lipophilic LAs and superior spreadability for outpatient dermatologic procedures and minor interventions.
- Hydrogels (useful): hydrogel plasters, patches, and dressings provide reliable surface-level anesthesia or analgesia, especially for chronic neuropathic or wound-related pain.
- Bigels (conceptual): potentially beneficial for dermal delivery when improved rheology or dual-phase release is desired.

#### 5.2.2. Mucosal and Cavity-Accessible Environments

- Organogels perform well under moderate hydration and adhere effectively to the mucosa, facilitating needle-free analgesia.
- Hydrogels also function reliably in moist mucosal settings when formulated as thermogels or mucoadhesive networks.
- Bigels may offer hybrid advantages, although mucosal performance remains speculative.

#### 5.2.3. Confined Soft-Tissue Planes (Oral, Dental, ENT, Buccal, Palatal)

- Hydrogels: excellent owing to their conformability and ability to localize after injection or thermogelation.
- Organogels: limited by hydration-induced destabilization.
- Bigels: conceptually attractive but unvalidated.

#### 5.2.4. Regional, Perineural, Peri-Incisional, or Deep Soft-Tissue Delivery

- Hydrogels dominate owing to their predictable injectability, retention, and resistance to motion and edema.
- Organogels are not suitable.
- Bigels lack any evidence of anatomical persistence or safety in deep tissues.

#### 5.2.5. Intra-Articular and Synovial Spaces

- Hydrogels are uniquely compatible with synovial turnover and biomechanical properties of joints.
- Organogels and bigels are not viable because of washout and phase instability.

#### 5.2.6. Implant-Adjacent or Surgical-Device Interfaces

- Hydrogels have demonstrated controlled release and tunable degradation in this domain.
- Organogels and bigels lack supporting data.

Overall, hydrogels occupy the broadest anatomical space, organogels naturally align with surface-directed applications, and bigels remain a hybrid concept without in vivo demonstration. This mapping establishes the clinical context for translational and regulatory comparisons in Section 5.3.

### 5.3. Translational Landscape and Regulatory Gradient

Translational maturity varies markedly among the gel platforms. The gradient is not a reflection of theoretical potential, but of reproducibility, manufacturing control, validated release behavior, and clinical evidence.

#### 5.3.1. Hydrogels—Most Advanced and Closest to Broad Clinical Use

- Approved products exist for dermatologic, mucosal, and wound care anesthesia.
- Investigational deep-tissue depots (perineural, intra-articular, and peri-incisional) show promising feasibility and early analgesic benefits.
- Regulatory needs include standardized pharmacokinetic endpoints, harmonized in vitro–in vivo correlations, and multicenter procedural trials.
- Manufacturing maturity is the highest, with validated sterilization pathways and consistent rheology [61,69,158,159,160].

#### 5.3.2. Organogels—Mechanistically Appropriate but Translationally Immature

- Compounding dominates clinical use, and no regulatory-approved LA organogel depots exist.
- Heterogeneous compositions (gelator purity, hydration, and solvent ratios) compromise reproducibility and batchwise pharmacokinetics.
- Clinical evidence is limited to small dermal/mucosal or chronic neuropathic pain studies, and procedural and interventional settings remain unexplored.
- Regulatory pathways are undefined owing to the lack of standardized manufacturing pipelines and controlled human trials [161,162].

#### 5.3.3. Bigels—Concept-Stage, No Validated Pharmacology

- No in vivo analgesic data or validated pharmacokinetic profiles exist for bigels used for LA delivery.
- Phase stabilization, interpenetrating network reproducibility, and sterilization feasibility remain unresolved.
- Their status reflects design promise, but no established translational trajectory [12,13,129].

Overall, hydrogels are the only platforms with validated surface-level regulatory pathways and emerging deep-tissue potential. Organogels are limited by the lack of reproducible formulations and controlled clinical studies. Bigels are exploratory biphasic constructs that require foundational pharmacological validation.

From a regulatory perspective, practical development pathways for gel-based LA depots differ substantially by platform and intended use. For hydrogels, the most practical near-term regulatory strategy involves incremental expansion from approved topical or mucosal products toward deeper or procedural applications using well-characterized polymers and established sterilization methods. Regulatory feasibility is enhanced by leveraging known local anesthetics, focusing on local pharmacokinetics and systemic exposure mitigation, and aligning early clinical endpoints with procedure-specific analgesic duration and safety profiles.

For organogels, translation will likely require a shift away from heterogeneous compounded formulations toward standardized compositions with defined gelators, solvent systems, and reproducible rheological properties. Practical regulatory progress depends on demonstrating batch-to-batch consistency, stability under physiologic hydration, and controlled release behavior in well-defined superficial indications, such as dermal or mucosal anesthesia.

Bigels currently lack a defined regulatory pathway for LA delivery. Their advancement will require foundational in vivo pharmacokinetic and safety studies to establish phase stability, release predictability, and local tolerability. In the near term, bigels are best positioned as exploratory formulation platforms rather than immediate clinical candidates, with regulatory development contingent upon first demonstrating reproducible biphasic behavior and scalable manufacturing control.

### 5.4. Practical Design and Selection Framework

Effective platform selection depends on integrating the matrix architecture, drug physicochemistry, tissue mechanics, workflow considerations, and regulatory feasibility. The question is not which depot class is “best,” but which platform is appropriate for a specific combination of anatomical sites, LA properties, and operational constraints [12,56,140,161,162,163,164].

#### 5.4.1. Tissue Mechanics and Depot Persistence

- Dynamic or hydrated environments (perineural, peri-incisional, intra-articular):

→ Hydrogel only

- Barrier-dense, superficial environments (skin, mucosa):

→ Organogel > Hydrogel patch

- Confined soft tissues:

→ Hydrogel; bigel = untested

- High moisture or irrigation (surgical fields):

→ Hydrogel, not organogel

#### 5.4.2. Drug Physicochemistry

- Lipophilic LAs in base form → Organogel or Bigel concept
- Ionized or amphiphilic LAs → Hydrogel
- High-dose extended analgesia with predictable pharmacokinetics → Hydrogel
- Need for multiphasic or combination-agent delivery → Hydrogel or Bigel prototypes

#### 5.4.3. Workflow and Clinical Operations

- Ambulatory, needle-free settings → Organogel
- Operating room, peri-incisional infiltration, deep blocks → Hydrogel
- Implant-adjacent → Hydrogel
- Outpatient dermatologic procedures → Organogel or Bigel concept

#### 5.4.4. Regulatory Feasibility and Development Pathway

- Near-term clinical translation → Hydrogel
- Early-phase formulation research → Organogel or Bigel
- High reproducibility/batch control required → Hydrogel only
- Novel dual-release or multi-agent systems → Bigel concept or multifunctional hydrogels

These principles emphasize that platform choice emerges from aligning matrix chemistry, anesthetic properties, and site-specific mechanics, rather than attempting to generalize a single optimal depot system. A consolidated platform-level comparison highlighting the architectural distinctions, release behavior, anatomical suitability, and translational readiness across hydrogels, organogels, and bigels is presented in Table 5.

## 6. Conclusions

Gel-based LA depots have advanced from empirical topical formulations to a diverse family of rationally engineered platforms capable of sustaining intratissue drug concentrations, reducing systemic exposure, and expanding the clinical range of LA therapies. Across this landscape, hydrogels, organogels, and emerging bigels represent three mechanistically distinct classes that are not interchangeable alternatives but complementary solutions positioned along different anatomical, physicochemical, and translational axes. From an evidence-level perspective, the maturity of supporting data differs substantially across platforms. Hydrogels are supported by the most robust preclinical and clinical evidence, including approved topical products and early procedural trials, whereas organogels rely primarily on exploratory and surface-oriented studies, and bigels remain at a conceptual or formulation-stage level without validated in vivo LA pharmacology. These differences necessitate cautious interpretation of comparative claims and underscore the importance of aligning platform selection with the strength and context of available evidence.

Hydrogels are the most mature and versatile polymeric matrices for LA delivery. Their aqueous, conformable networks allow predictable injectability, tunable degradation, and compatibility with both hydrophilic and amphiphilic anesthetics. Preclinical studies across perineural, peri-incisional, soft-tissue, and intra-articular models consistently demonstrated localized retention, attenuated systemic peaks, and multi-day analgesia, whereas early clinical trials in perioperative and interventional settings showed promising feasibility. Simultaneously, hydrogels maintain a strong role in chronic and outpatient care through established topical and mucosal products. Their regulatory readiness, batchwise reproducibility, and well-characterized safety position make hydrogels the leading platform for the broad clinical deployment of gel-based LA depots, with established surface-level products and the most advanced trajectory toward deep-tissue applications.

Organogels, in contrast, provide lipid-rich, surface-aligned matrices optimized for solubilizing neutral or lipophilic LA bases and facilitating dermal, transdermal, and mucosal penetration. Their favorable spreadability and barrier matching make them intuitive for needle-free, outpatient topical analgesia, and preclinical studies have confirmed prolonged lidocaine action with reduced systemic uptake. However, clinical evidence is limited to small trials and compounded preparations used mainly for chronic neuropathic or focal musculoskeletal pain. The lack of standardized formulations, batch variability, and minimal procedural or perioperative testing define a translational gap that restricts organogels to chronic/outpatient topical niches rather than deep-tissue analgesia.

Bigels, as biphasic hybrids integrating hydrogel and organogel domains, offer a conceptual bridge combining lipid-phase solubilization with aqueous-phase rheological reinforcement, mucoadhesion, and potentially multiphasic release. Although mechanistically promising, bigels remain preclinical- and formulation-stage constructs, without validated pharmacokinetics or analgesic performance in vivo. Their future utility depends on overcoming phase stabilization, sterilization, and scalability bottlenecks while generating foundational LA-specific data.

These platforms define a multidimensional design space in which no single depot is universally optimal. Instead, platform selection should align with (1) the physicochemical form of the LA (base vs. salt), (2) the anatomical environment (skin, mucosa, soft-tissue planes, intra-articular spaces), (3) procedural versus outpatient goals, and (4) regulatory and manufacturing considerations. Hydrogels currently offer the broadest applicability, including perioperative regional and infiltrative anesthesia, whereas organogels remain best suited for outpatient dermatologic, mucosal, and chronic neuropathic use. Bigels represent an emerging frontier for hybrid surface-directed systems once foundational in vivo pharmacology is established.

Progress in crosslink chemistry, stimuli-responsive linkers, lipid–polymer hybridization, and machine-learning-guided formulation design is likely to drive a new generation of smart, tunable depots capable of adaptive, feedback-responsive analgesia. Equally important will be standardized pharmacokinetic–pharmacodynamic assays, harmonized release-testing methodologies, and large-scale procedural trials that clarify where gel depots provide meaningful incremental benefits over existing single-shot, catheter-based, or topical LA strategies.

Across both procedural and outpatient care, gel-based depots offer a pathway toward safer, longer-acting, and workflow-compatible LA delivery. Continued integration of mechanistic materials science with clinically anchored study designs is essential for translating hydrogels, organogels, and bigels into next-generation anesthetic platforms that bridge acute perioperative needs and chronic pain management.

## Figures and Tables

**Table 1:** Scope and evidence coverage of studies included in this narrative review on gel-based local anesthetic delivery.

Platform/Domain	Evidence Type Included	Representative Models or Contexts	Rationale for Inclusion
Hydrogels—deep-tissue delivery	Preclinical studies and early-phase clinical investigations	Perineural and regional nerve block models; peri-incisional and soft-tissue infiltration; intra-articular and synovial environments; implant-adjacent depots	Strong mechanistic rationale and the most advanced translational evidence for sustained, localized LA delivery
Hydrogels—topical and mucosal applications	Approved clinical products, randomized trials, and prospective clinical studies	Lidocaine plasters, wound dressings, oral and dental gels	Established regulatory pathways and well-characterized pharmacokinetics supporting surface-level anesthesia and analgesia
Organogels—dermal and transdermal delivery	Preclinical studies and limited controlled human evaluations	Dermal, transdermal, and mucosal barrier models; chronic neuropathic or focal musculoskeletal pain	Barrier-aligned lipid matrices optimized for lipophilic LAs and needle-free outpatient use
Organogels—compounded clinical formulations	Observational studies, crossover trials, and clinical case series	Compounded PLO formulations used in chronic pain practice	Illustrates real-world feasibility while highlighting formulation heterogeneity and translational limitations
Bigels—LA–specific	None or very limited	—	No validated in vivo pharmacokinetic or analgesic data currently available for LA delivery
Bigels—supporting non–LA studies	Preclinical formulation and proof-of-concept studies	Biphasic hydrogel–organogel systems loaded with NSAIDs or antimicrobials	Provides indirect mechanistic support for biphasic gel architectures relevant to future anesthetic applications

Abbreviations: LA, local anesthetic; NSAID, non-steroidal anti-inflammatory drug; PLO, pluronic-lecithin organogel.

**Table 2:** Hydrogel design strategies for local anesthetic delivery.

Hydrogel Design Strategy	Representative Materials/Platforms	Primary Release/Control Mechanism	Mechanical/Anatomical Profile	Advantages of Local Anesthetic Delivery	Key Limitations/Considerations
Thermoresponsive hydrogels	Poloxamer 407; PLGA–PEG–PLGA; PNDJ; PNIPAAm-based copolymers	Temperature-triggered sol–gel transition; diffusion-dominated release	Conformable in situ gelation; suitable for perineural, peri-incisional, intra-articular spaces	Workflow compatibility; extended residence; attenuated systemic peaks	Dilution-induced erosion; limited stiffness; storage and thermal instability; burst release risk
Covalently crosslinked and dual-network hydrogels	HA–poloxamer hybrids; gelatin–tyramine; NHS–PEG–NHS; chemically crosslinked PEG or polysaccharides	Diffusion through dense mesh plus gradual covalent or enzymatic cleavage	Mechanically reinforced; suitable for deep soft tissue, perineural, and implant-adjacent sites	Improved depot integrity; reproducible release; tunable stiffness and degradation	Complex synthesis; potential delayed onset if too dense; resorption balance needed
Stimuli-responsive hydrogels	pH-labile linkers; thioketal or boronic ester ROS-responsive motifs; MMP-cleavable peptides	Context-sensitive degradation driven by acidosis, oxidative stress, or protease activity	Designed for inflamed, ischemic, or remodeling tissues such as wounds or joints	Synchronizes anesthetic exposure with biological cues; minimizes unnecessary dosing	High inter-patient variability in triggers; risk of under/over-release; sensitive manufacturing
Multifunctional co-delivery hydrogels	Hydrogels carrying dexmedetomidine, NSAIDs, antimicrobials, antioxidants, peptides	Combined diffusion and degradation release of multiple agents	Applicable to wounds, peri-incisional infiltration, regional blocks	Multimodal analgesia; anti-inflammatory or regenerative synergy	Formulation complexity; potential drug–drug interactions; regulatory challenge
Composite and particle-reinforced hydrogels	Hydrogels with liposomes, mesoporous silica, polymeric nanoparticles, graphene, or responsive fillers	Hierarchical diffusion barriers; multiphasic release; external or internal stimulus response	Enhanced strength and tunability; suitable for high-strain environments	Controlled early and late phases; on-demand or feedback-modulated analgesia	QC complexity; biocompatibility of fillers; limited clinical experience

Abbreviations: HA, hyaluronic acid; MMP, matrix metalloproteinase; NHS, N-hydroxysuccinimide; NSAID, non-steroidal anti-inflammatory drug; PEG, polyethylene glycol; PLGA, poly(lactic-co-glycolic acid); PNDJ, poly(N-isopropylacrylamide)-based triblock copolymer; PNIPAAm, poly(N-isopropylacrylamide); QC, quality control; ROS, reactive oxygen species.

**Table 3:** Clinical hydrogel-based local anesthetic products and investigational depots.

Platform/Product	Matrix Type	Local Anesthetic(s)	Clinical Context/Route	Key Clinical Outcomes	Safety Profile	Reference
Lidoderm^®^/Versatis^®^ (5% lidocaine plaster)	Hydrogel adhesive patch	Lidocaine	Dermal; chronic neuropathic pain	Reduced allodynia; improved pain scores; <0.3 μg/mL systemic absorption	Excellent tolerability; minimal sensory block	[63,64,65,66]
Astero^®^ (TRI-726, 4% lidocaine)	Hydrogel wound dressing	Lidocaine	Acute and chronic wounds	Multi-day pain reduction; moisture compatibility	No device-related complications	[67,68]
Regenecare^®^ HA	HA–collagen hydrogel dressing	Lidocaine	Chronic or contaminated wounds	Local analgesia; improved wound environment	Good compatibility	[69]
MicroLyte^®^ Ag/Lidocaine	Silver–polymer composite thin film	Lidocaine	Wound care; postsurgical incisions	Analgesia plus antimicrobial benefits	No significant adverse events	[70]
Oraqix^®^ (2.5% lidocaine + 2.5% prilocaine)	Thermogelling oral hydrogel	Lidocaine + prilocaine	Intraoral mucosal anesthesia	Rapid onset; effective procedural anesthesia	Minimal systemic absorption	[71]
Dentipatch^®^ (20% lidocaine film)	Mucoadhesive hydrogel patch	Lidocaine	Oral mucosal needle-site anesthesia	Predictable transmucosal delivery	Well tolerated	[72]
PF72–ropivacaine thermogel	Thermoresponsive poloxamer–HA hydrogel	Ropivacaine	Peri-incisional infiltration	72 h analgesia; reduced opioid use	No device-related adverse events	[73,74,75]
Welpass (poloxamer–alginate hybrid)	Injectable thermogel	Ropivacaine	Bariatric surgery infiltration	Non-inferior to continuous infusion	No complications reported	[76]
Poloxamer 407 ropivacaine gel	Preformed hydrogel depot	Ropivacaine	Thoracoscopic and laparoscopic surgery	72 h analgesia similar to paravertebral or wound catheters	No impairment of wound healing	[77,78]
Sodium–CMC ropivacaine gel	Viscous biodegradable hydrogel	Ropivacaine	Pediatric reconstructive donor-site analgesia	Improved early pain control; reduced PCA demand	No adverse healing issues	[79]
Pre-incisional 5% lidocaine hydrogel plaster	Topical hydrogel patch (repurposed)	Lidocaine	Craniotomy peri-incisional anesthesia	Modest benefit in selected subgroups	Excellent tolerability	[80]
Gelatin-based bupivacaine-eluting pedicle screw ring	Implant-integrated hydrogel	Bupivacaine	Spinal surgery	Multi-day localized release; reduced systemic peaks	Favorable histology	[81]

Abbreviations: CMC, carboxymethylcellulose; HA, hyaluronic acid; PCA, patient-controlled analgesia.

**Table 4:** Organogel and bigel platforms for local anesthetic delivery: Preclinical and clinical evidence.

Evidence Level	Platform/Model	Representative Composition	Anesthetic/Probe	Key Findings	Advantages	Limitations	Reference
Preclinical	Dermal organogels	Lecithin–isopropyl myristate; lanolin–poloxamer hybrid; lipid–poloxamer nanostructured organogels	Lidocaine	Prolonged dermal anesthesia; reduced flux from ~17 to ~12 μg/cm^2^/h; improved rheology; favorable cytocompatibility	High solubilization of lipophilic LAs; strong barrier alignment; sustained cutaneous action	Dermal-only relevance; limited data for bupivacaine/ropivacaine; few behavioral models	[131]
Preclinical	Transdermal organogels	Lecithin–isopropyl palmitate; PLO; permeation enhancers (oleic acid, terpenes)	Lidocaine	Enhanced skin retention; modulated stratum corneum fluidization; sustained release across ex vivo skin	Needle-free delivery; tunable permeation; improved patient comfort	High variability; permeation enhancer safety concerns; limited in vivo PK	[132]
Preclinical	Mucosal organogels	Thermoreversible PEG–poloxamer systems; PEG4000; Sacha inchi oil	Lidocaine	72 h release in artificial saliva; prolonged analgesia vs. hydrogel and solution; strong mucoadhesion	Stable under high moisture; enhanced retention; ideal for oral/mucosal analgesia	Limited datasets; no perioperative mucosal models	[133]
Preclinical (mechanistic)	Barrier mechanistic studies	PLO; lanolin-based gels	Non-anesthetic probes (e.g., NSAIDs, dyes)	Clarified barrier transport, flux modulation, lipid phase interactions	Provides mechanistic insight into organogel behavior	Not directly anesthetic-focused; extrapolation required	[134,135]
Clinical	PLO-based lidocaine organogel	Lecithin–isopropyl myristate + poloxamer aqueous phase (PLO)	5% lidocaine	Double-blind crossover trial: modest pain reduction but similar to placebo; good tolerability	Feasible human use; safe topical analgesia	Limited efficacy; no procedural/perioperative data; compounded variability	[136]
Clinical (observational)	Compounded organogels	Lidocaine ± ketamine/baclofen/amitriptyline mixtures in PLO	Lidocaine ± adjuncts	Used for focal neuropathic or musculoskeletal pain; evidence from case series only	Customizable; accessible in practice	No PK data; no controlled trials; inconsistent formulations	[134]
Conceptual/preclinical (non-LA)	Bigels	Interpenetrating hydrogel + organogel networks; biphasic lipid–aqueous matrices	Ibuprofen, metronidazole (non-LA)	Improved rheology; enhanced deposition; smoother biphasic controlled release	Combines strengths of hydrogel + organogel; theoretical LA suitability	No in vivo LA data; no PK; unvalidated for analgesia	[137,138]

Abbreviations: LA, local anesthetic; NSAID, non-steroidal anti-inflammatory drug; PEG, polyethylene glycol; PK, pharmacokinetics; PLO, pluronic–lecithin organogel.

**Table 5:** Platform-level comparison of hydrogel, organogel, and bigel depots for local anesthetic delivery.

Criterion	Hydrogels	Organogels	Bigels
Matrix architecture	Hydrated crosslinked or physically associated polymer network (aqueous continuous phase)	Lipid or semi-polar solvent structured by LMWG supramolecular assembly	Biphasic hybrid with interpenetrating aqueous (hydrogel) and lipid (organogel) domains
LA compatibility	Hydrophilic and amphiphilic LAs; many lipophilic LAs via mesh entrapment; suitable for responsive linkers	High solubility for neutral/lipophilic LAs; strong barrier partitioning	Conceptual dual-phase solubilization (lipophilic in lipid phase; hydrophilic excipients in aqueous phase)
Primary release mechanism	Diffusion plus degradation (hydrolytic, enzymatic, ROS/pH-responsive)	Diffusion through lipid matrix; barrier-modulated transport	Theoretical biphasic or multiphasic release; not yet validated in vivo
Mechanical/anatomical suitability	Stable in dynamic or hydrated tissues (perineural, peri-incisional, joint, soft-tissue planes)	Best suited for dermal, transdermal, or mucosal surfaces; unstable in deep, hydrated tissue environments	Improved rheology vs. organogels but lacking any deep-tissue validation
Regulatory maturity	Multiple approved products (topical, mucosal, wound); injectable depots investigational	No approved LA organogels; mostly compounded preparations	Preclinical formulation-stage only; no PK or analgesic data
Manufacturing & QC	Established sterilization methods; consistent rheology; scalable	High batch variability (gelator purity, hydration, solvent ratios)	Dual-phase QC complexity; no standardized frameworks
Strengths	Conformable, injectable, mechanically robust, adaptive release capability	Excellent solubilization of lipophilic LAs; strong barrier matching; needle-free delivery	Integrates hydrogel and organogel advantages; potential multiphasic release and multi-agent design
Limitations	Sterilization challenges for responsive linkers; risk of burst release	Formulation heterogeneity; limited to superficial tissues; limited clinical evidence	No validated in vivo analgesia; phase stability and sterilization unresolved
Ideal use cases	Regional blocks, peri-incisional infiltration, intra-articular dosing, implant-adjacent release	Outpatient dermal, transdermal, or mucosal anesthesia; chronic focal pain	Exploratory hybrid topical systems; early-stage formulation research

Abbreviations: LA, local anesthetic; LMWG, low-molecular-weight gelator; PK, pharmacokinetics; QC, quality control; ROS, reactive oxygen species.

## Data Availability

No new data were created or analyzed in this study. Data sharing is not applicable to this article.

## Abbreviations

The following abbreviations are used in this manuscript:

LA	Local anesthetic
ROS	Reactive oxygen species
LMWG	Low-molecular-weight gelator
PNDJ	Poly(ethylene glycol)-poly(N-isopropylacrylamide)-poly(ethylene glycol)
HA	Hyaluronic acid
MMP	Matrix metalloproteinase
NSAID	Non-steroidal anti-inflammatory drug
PLO	Pluronic–lecithin organogel
